# Traumatic Brachial Plexus Root Avulsion With Pseudomeningocele: A Case Series

**DOI:** 10.7759/cureus.55408

**Published:** 2024-03-02

**Authors:** Praveen K Sharma, Chakradhar Ravipati, Vinoth Pandian, Sakthi Ganesh Subramonian, Karpagam RK

**Affiliations:** 1 Department of Radiodiagnosis, Saveetha Medical College and Hospital, Saveetha Institute of Medical and Technical Sciences (SIMATS) Saveetha University, Chennai, IND

**Keywords:** pseudomeningocele, trauma, magnetic resonance imaging, traction, brachial plexus

## Abstract

Traumatic avulsion pseudomeningocele of the brachial plexus is an uncommon and challenging condition with particular diagnostic and treatment challenges. This case series intends to investigate the unusual consequences of brachial plexus damage, emphasizing the significance of surgical procedures and rehabilitation strategies. Three cases of traumatic avulsion pseudomeningocele with medical histories, imaging studies, procedures, and recovery plans were carefully examined. The rehabilitation approaches and surgical procedures are outlined in detail. Each case had its own unique set of difficulties and complications. Nerve grafting and pseudomeningocele repair surgery were performed. The outcomes were evaluated based on neurological examination, range of motion, sensory recovery, and patient reports. Only a few patients showed discernible improvements in their quality of life, motor function, and discomfort. In this case series, we highlight the people with traumatic avulsion pseudomeningocele of the brachial plexus and recount their inspiring journeys. Surgical procedures and rehabilitation approaches have produced favorable outcomes regarding recovering functionality and enhancing patients' general well-being. These results highlight the value of interdisciplinary partnerships and individualized strategies in treating this uncommon illness. Further, more profound research and long-term follow-up are required regarding the condition and optimizing the treatment methods for this challenging clinical entity.

## Introduction

Spinal cord herniation is classified as posttraumatic, spontaneous, and iatrogenic herniations. A complete tear of one or more spinal nerve roots constitutes nerve root avulsion [1]. High-energy trauma like motor vehicle collisions leads to avulsion injuries [2]. Traumatic avulsion pseudomeningocele of the brachial plexus is an uncommon and challenging disorder that presents severe difficulties for patients and medical professionals. Traumatic avulsion injuries to the brachial plexus can cause severe functional impairments, such as loss of motor function, sensory deficiency symptoms, and persistent discomfort. Avulsion pseudomeningocele, a condition that causes the breakdown of nerve roots and the development of a meningeal cyst, complicates the clinical picture. Traction injuries from motorcycle accidents lead to a complete avulsion of the intradural, preganglionic brachial plexus. Following complete avulsion of the brachial plexus, pseudomeningocele evolves due to cerebrospinal fluid (CSF) accumulation in the damaged meninges that cover the injured nerve roots. Neurological deficits or partial motor function with a nerve root avulsion are repaired surgically [3]. Magnetic resonance imaging (MRI), physical exams, and nerve conduction studies to localize the injury determine the extent of the neurological deficit [4]. Avulsion of the brachial plexus can be associated with a vascular injury (subclavian artery or vein), a complicated entity to manage. Traumatic posterior pseudomeningocele entails a problematic surgical approach. It is essential to comprehend the difficulties that patients with traumatic avulsion pseudomeningocele address to create efficient treatment regimens and enhance patient outcomes. This study intends to add to the body of information, inspire additional investigation, and progress the field by investigating the experiences of patients undergoing surgical interventions and rehabilitation.

## Case presentation

Case presentation 1

A 21-year-old male presented to the neurology department with right upper limb weakness following a traumatic event three weeks prior, resulting in injuries to his right shoulder, arm, and forearm. Upon neurological examination, there was observed loss of function at the shoulder, arm, elbow, and forearm, characterized by weak flexion and normal extension at the wrist joint and a notable loss of sensation throughout the affected right upper limb in the form of absence of tactile and proprioceptive sensation, further complicating the clinical picture.

**Figure 1:**
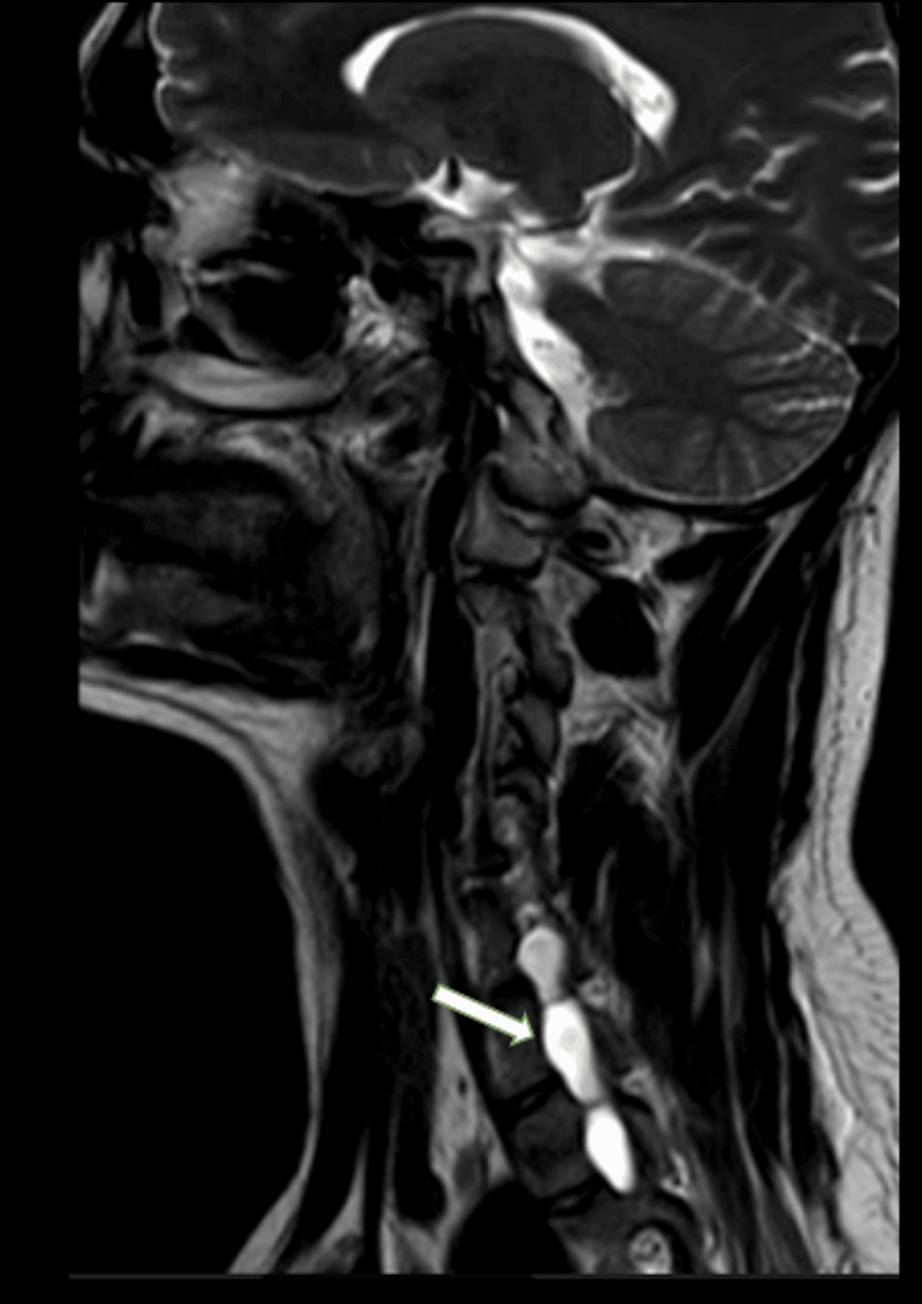
MRI-brachial plexus T2 right parasagittal section of case 1 MRI: Magnetic resonance imaging T2 right parasagittal section of the MRI-brachial plexus shows extradural, cystic formations that resemble cerebrospinal fluid collections within the spinal canal as indicated by the large thick white arrow. These collections are visible on both the dorsal and ventral aspects close to the spinal cord, extending through the right neural foramina at the levels of C6-C7, C7-T1, and T1-T2 vertebrae. Notably, these formations lack central neural elements and are bordered by smooth to lobulated margins.

**Figure 2:**
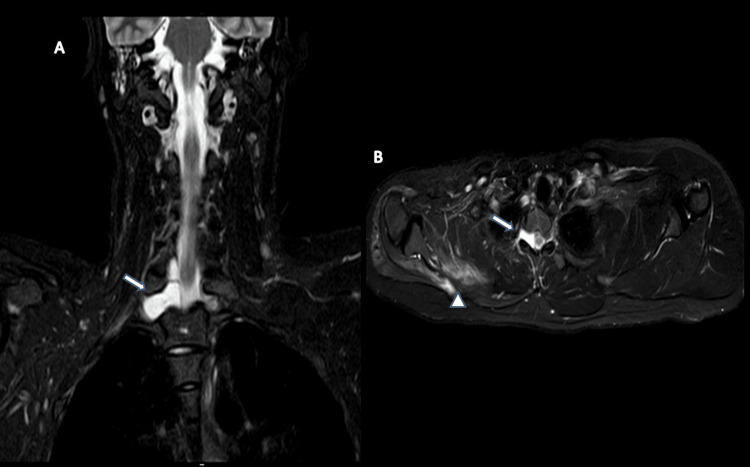
MRI-brachial plexus (A) STIR coronal section, (B) STIR axial section of case 1 MRI: Magnetic resonance imaging; STIR: short tau inversion recovery; CSF: cerebrospinal fluid MRI of the brachial plexus (A) STIR coronal section showing extradural, CSF-filled collection extending to the right neural foramina at the levels of the T1-T2 vertebrae, indicated by a large white arrow. (B) STIR axial section shows a CSF-filled structure extending into the right neural foramen at the levels of the C7-T1 vertebrae, indicated by a large thick white arrow. Shoulder and chest wall muscles, including the supraspinatus, teres, subscapularis, deltoid, pectoralis major and minor, and trapezius, shows signs of mild atrophy compared to the contralateral side accompanied by fatty infiltration and edema-like characteristics, highlighted by a small thick white arrowhead.

MRI of the brachial plexus revealed the presence of extradural, multifocal cystic lesions resembling CSF collections within the spinal canal (Figure 1). These were located on the right side, both dorsal and ventral aspects close to the spinal cord, and extended to the right neural foramina at the levels of C7-T1 and T1-T2 vertebrae (Figure 2A, 2B). The lesions had an absence of central neural elements and peripheral margins that were smooth to lobulated, causing mild compression and slight leftward displacement of the cervical and dorsal cord without any signs of myelomalacia. Additionally, mild atrophy with fatty infiltration and edema-like changes were noted in the right shoulder muscles (including the supraspinatus, infraspinatus, teres minor, subscapularis, and deltoid) and the visualized anterior and posterior chest wall muscles (pectoralis major and minor and trapezius), as shown in Figure 2B.

Electroneuromyography of the right shoulder and upper limb indicated motor and sensory deficits in the respective nerves and muscles. The findings led to a diagnosis of traumatic avulsion pseudomeningocele of the right brachial plexus, resulting in chronic brachial plexopathy and muscle atrophy with fatty infiltration and chronic denervation.

The patient underwent surgical exploration, which revealed pre- and postganglionic avulsion injuries of the right C5, C6, C7, C8, and T1 nerve roots, and spinal cord protrusions were observed through the dural defects with a direct connection between the CSF in the spinal subarachnoid space and the pseudomeningoceles were seen. To prevent the spinal cord from herniating again, the pseudomeningoceles were primarily closed, followed by a dural patch repair. The innovative surgical approach for nerve dysfunction involved transferring intercostal nerves T2 to T6 to the affected areas, aiming to restore both motor and sensory functions to the right upper limb. Postoperatively, the patient was enrolled in an intensive physiotherapy regimen, incorporating neuromuscular electrical stimulation to combat muscle atrophy, range-of-motion exercises to ensure joint flexibility, strength training to rebuild muscle capability, and sensory re-education to adapt to new nerve inputs.

Throughout the recovery process, closely monitored through regular follow-up visits, the patient demonstrated notable improvements. By six months, there was a significant increase in muscle strength, enabling partial flexion and extension at the elbow and wrist, alongside enhanced tactile sensations. Sensory adaptation, although full pre-injury strength levels were not yet achieved. The treatment team then tailored the rehabilitation program toward advanced strength and functional skills rehabilitation, aiming for continuous improvement.

Case presentation 2

A 28-year-old male who experienced a loss of movement and sensation in his left upper limb following a road traffic accident and sustaining an injury to the left side of his neck one week prior presented to the neurology department. The neurological evaluation revealed a complete lack of muscle strength, rated as 0 out of 5, in all muscle groups of the left arm, while sensation remained preserved and pulse strength was consistently found to be normal at 2+ across all evaluated sites. Diagnostic imaging and tests were conducted to assess the extent of the injury and determine a course of treatment.

**Figure 3:**
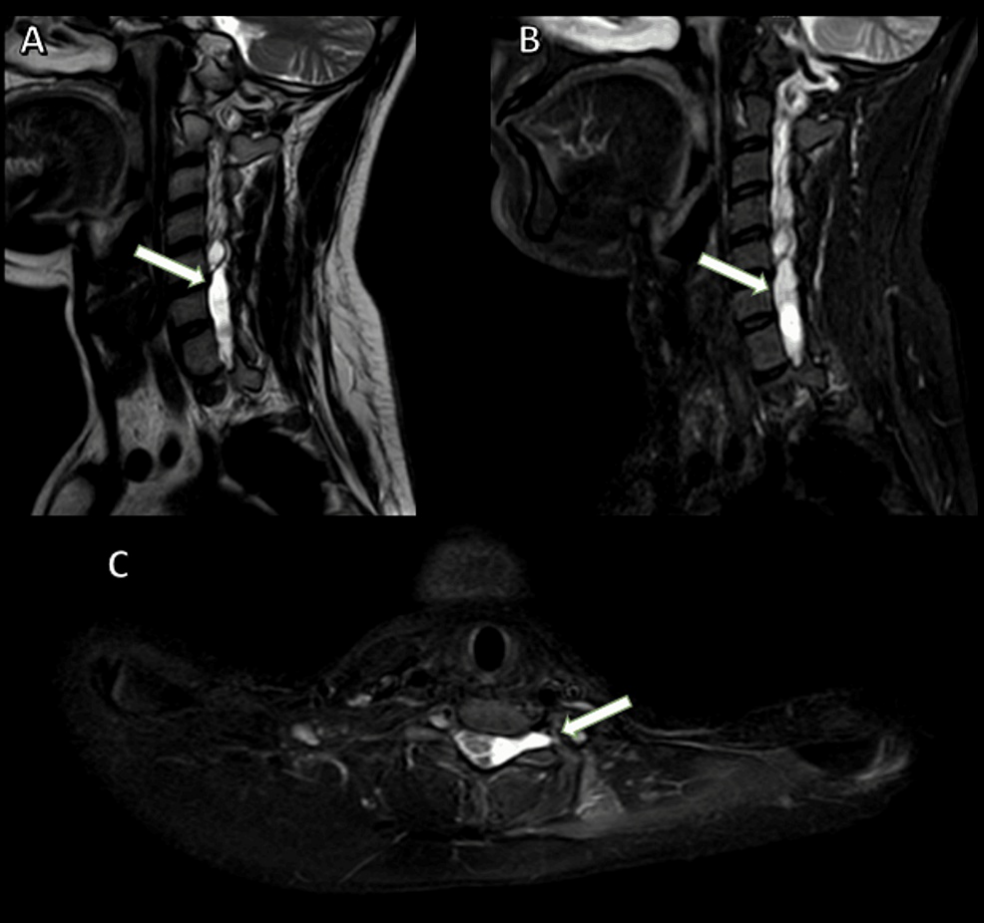
MRI-brachial plexus (A) T2 left parasagittal section, (B) STIR left parasagittal section, (C) STIR axial section of case 2 MRI: Magnetic resonance imaging; STIR: short tau inversion recovery; CSF: cerebrospinal fluid MRI of the brachial plexus (A) T2-weighted left parasagittal view, (B) STIR left parasagittal view showing extradural collections that resemble the CSF within the spinal canal, located laterally on the left side at the levels of the C4-C5, C5-C6, and C6-C7 vertebrae. These collections do not contain central neural elements and are bordered by margins that transition from smooth to lobulated, creating a significant mass effect and displacing the cervical cord laterally to the right. (C) STIR axial view, showing CSF-filled structure extending into the left neural foramen at the levels of the C6-C7 vertebrae, indicated by a large thick white arrow.

**Figure 4:**
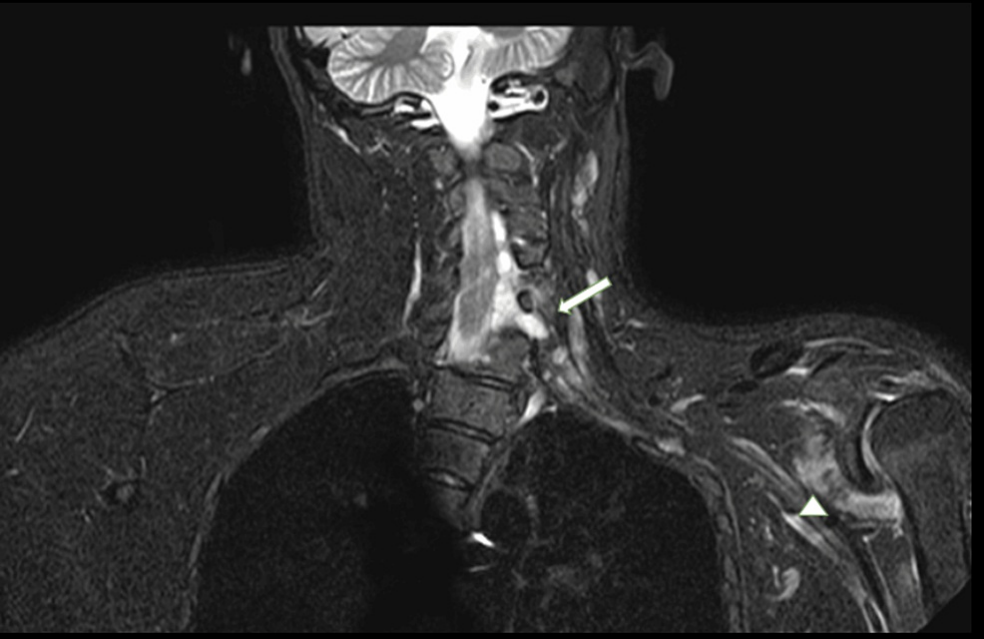
MRI-brachial plexus STIR coronal section of case 2 MRI: Magnetic resonance imaging; STIR: short tau inversion recovery; CSF: cerebrospinal fluid MRI-brachial plexus STIR coronal section showing extradural, CSF-filled collections in the spinal canal (left laterally) (at the levels of C4-C5, C5-C6, and C6-C7 vertebrae) with extension of the CSF-filled structure into the left neural foramen at the levels of C6-C7 vertebrae (large thick white arrow). The left brachial plexus (superior, middle, and inferior trunks in interscalene triangle, six divisions in costoclavicular space, lateral, posterior, and medial cords in retropectoralis minor space) appears as mild edematous (small thick white arrow head).

MRI of the brachial plexus revealed extradural, multifocal cystic-like collections resembling CSF within the spinal canal, positioned laterally on the left side at the levels of the C4-C5, C5-C6, and C6-C7 vertebrae (Figure 3A-3C). These collections were noted to have no central neural elements and smooth to lobulated margins, exerting a significant mass effect and displacing the cervical cord to the right, albeit without evidence of myelomalacia changes. Furthermore, there was an extension of the CSF-filled structures into the left neural foramen at the C6-C7 levels (Figures 3C, 4). Edema-like changes were also visible in the shoulder muscles (including the supraspinatus, infraspinatus, teres minor, subscapularis, and deltoid) and the left brachial plexus (superior, middle, and inferior trunks in inter-scalene triangle, six divisions in costoclavicular space, lateral, posterior, and medial cords in the retropectoralis minor space as represented in Figure 4 (small thick white arrow head).

Electroneuromyography of the left shoulder and upper limb confirmed motor and sensory deficits in the respective nerves and muscles. Based on these clinical findings, imaging results, and electroneuromyography data, a diagnosis of traumatic avulsion pseudomeningocele of the left brachial plexus was made, leading to brachial plexopathy and denervation.

The comprehensive and multidisciplinary treatment plan for this patient began with a critical surgical intervention. The surgical team performed closure of the pseudomeningocele to prevent spinal cord reherniation followed by a nerve transfer surgery using the left T1, T2, and T3 intercostal nerves to address the pre- and postganglionic injuries of the left C5, C6, and C7 nerves. This procedure aimed to reroute functional nerves to the muscles that had lost their original nerve supply. Postoperative care emphasized pain management, wound care, and prevention of secondary complications, leading to an uneventful recovery and subsequent discharge with comprehensive home care instructions.

Rehabilitation started two weeks postsurgery, focusing initially on passive exercises to maintain joint mobility and gradually incorporating active exercises for muscle strengthening and range of motion improvement. Electrical stimulation therapy was also used to facilitate muscle re-education and nerve regeneration. Remarkable progress was observed in the following months, with significant improvements in muscle strength, sensation, and functional use of the left upper limb.

Electroneuromyography six months postsurgery showed improved nerve conduction, indicative of successful nerve regeneration. By the one-year mark postsurgery, the patient had regained substantial function, able to perform complex tasks requiring fine motor skills, despite some remaining deficits in full shoulder range of motion.

Case presentation 3

A 40-year-old female, suffering from right upper limb weakness since a road traffic accident four months ago, presented to the neurology department. Neurological assessment highlighted profound muscle power loss at the shoulder, elbow, and proximal radio-ulnar joints, signifying severe functional impairment. Contrastingly, wrist flexion and extension remained relatively intact, suggesting a selective neurological impact that spared distal limb functions. Sensory evaluation was also conducted to gauge the extent of sensory nerve damage, examining touch, pain, temperature, and proprioception, which remained intact. Considering this complex neurological presentation diagnostic imaging, tests were conducted to assess the extent of the injury and determine a course of treatment.

**Figure 5:**
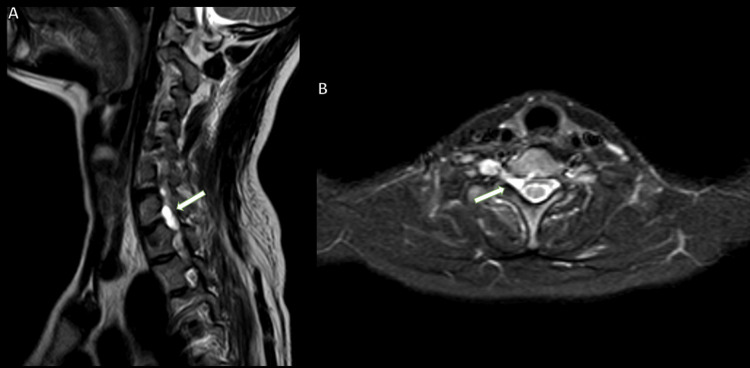
MRI-brachial plexus (A) T2 sagittal section, (B) STIR axial section of case 3 MRI: Magnetic resonance imaging; STIR: short tau inversion recovery; CSF: cerebrospinal fluid MRI-brachial plexus (A) T2 sagittal section shows the presence of extradural, multifocal collections that closely resemble the CSF within the spinal canal, situated on both the right dorsal and ventral sides. (B) STIR axial section shows these collections extend into the right neural foramen at the levels between the C5-C6 and C7-T1 vertebrae. Notably, these collections lack central neural elements and are characterized by margins that transition from smooth to lobulated. This results in mild compression and a slight leftward displacement of the cervical cord segments (large thick white arrow).

MRI of the brachial plexus indicated the presence of extradural, multifocal cystic formations that resemble the CSF within the spinal canal. These formations were located on the right dorsal and ventral aspects, extending to the right neural foramen across the vertebrae levels of C5-C6 and C7-T1 (Figure 5A, 5B). The findings noted an absence of central neural elements within these collections and peripheral margins that ranged from smooth to lobulated. These characteristics caused mild compression and a slight leftward displacement of the cervical and dorsal cord, without any evidence of myelomalacia changes.

**Figure 6:**
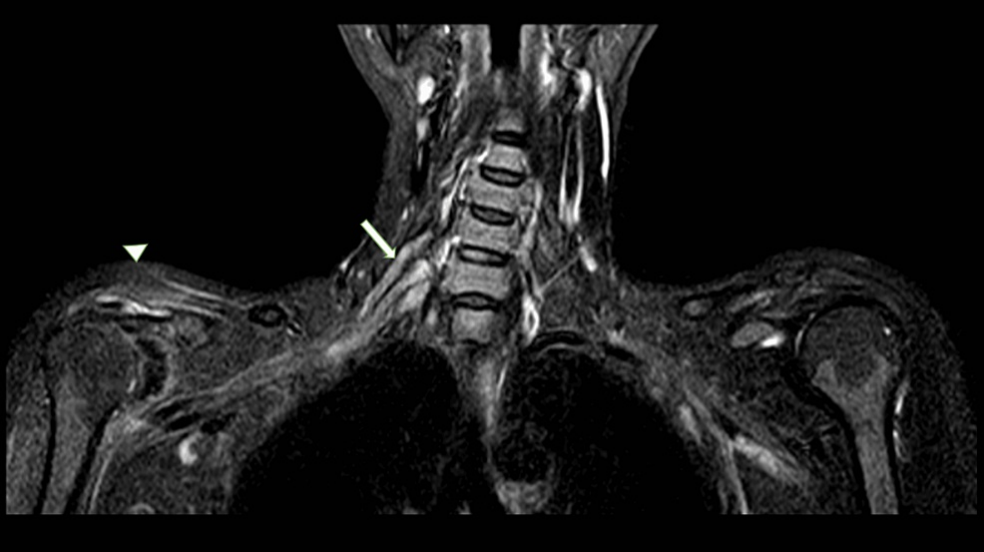
MRI-brachial plexus STIR coronal section of case 3 MRI: Magnetic resonance imaging short tau inversion recovery MRI of the brachial plexus using the STIR coronal section showing edematous the superior, middle, and inferior trunks situated within the interscalene triangle, as indicated by a large white arrow. Additionally, the shoulder muscles (comprising the supraspinatus, infraspinatus) show mild atrophic changes accompanied by fatty infiltration, highlighted by small thick white arrowheads (top of form).

Further imaging of the right brachial plexus, which includes the superior, middle, and inferior trunks within the inter-scalene triangle, six divisions in the costoclavicular space, and lateral, posterior, and medial cords in the retropectoralis minor space, revealed mild edematous changes (Figure 6, long white arrow). Mild atrophy with fatty infiltration is seen in the shoulder muscles (supraspinatus, infraspinatous) as shown in Figure 6 (short white arrow head).

Electroneuromyography of the right shoulder and upper limb confirmed motor and sensory deficits in the respective nerves and muscles. Based on the clinical examination, MRI findings, and electroneuromyography results, a diagnosis of traumatic avulsion pseudomeningocele of the right brachial plexus was made. This condition led to chronic brachial plexopathy, muscle atrophy with fatty infiltration, and chronic denervation.

Treatment involving surgical exploration and rehabilitation was undertaken. The surgery revealed pre- and postganglionic injuries across the right C5 to T1 nerves alongside spinal cord protrusions through dural defects that facilitated a direct link between the CSF in the spinal subarachnoid space and pseudomeningoceles. To mitigate the risk of future spinal cord herniation, an initial step of closing the pseudomeningoceles was taken, succeeded by the application of a dural patch for repair. The treatment strategy also included a nerve transfer procedure utilizing nerves from the right T2 to T6 intercostal regions, aimed at reestablishing motor function by redirecting functioning nerves to the compromised areas. Following the surgical intervention, a rigorous physiotherapy program was initiated, concentrating initially on managing pain and enhancing mobility before progressively introducing strength and coordination exercises.

Significant improvements in motor capabilities were noted within the first six months, with steady advancement observed during subsequent checkups. A year into the treatment, the patient achieved considerable functional improvements, marking a milestone in regaining independence for daily tasks. Remarkable recovery continued into the second year posttreatment, with the patient showcasing the ability to undertake intricate tasks despite some persisting limitations in movement.

## Discussion

The reported case series provides insight into the uncommon and challenging syndrome known as traumatic avulsion pseudomeningocele of the brachial plexus. This series offers essential insights into the diagnosis, treatment, and outcomes of people with this complicated ailment by looking at multiple examples. The brachial plexus (nerves C5, C6, C7, C8, and T1) controls the motor and sensory functions of the upper extremities. The high-force accident leads to a brachial plexus injury due to the cervical spine's biomechanics and motion range. Brachial plexus injury mainly occurs in young adults between 20 and 30 years following motorcycle accidents. Brachial plexus injury is due to stretching, tearing, bruising, hematoma, or a foreign body leading to avulsion pseudomeningocele. Brachial plexus avulsion is often associated with fractures or hematoma, aggravating the prognosis and delaying treatment. Pseudomeningocele is associated with avulsion in 80% of cases. The pathophysiology of traumatic brachial plexus injuries is crucial, as the clinical history and clinical presentation dictate the evaluation and treatment. Automobile accidents result in forcible distraction of the arm and contralateral hyperflexion of the neck, which strains the nerves and causes neuropraxia, dura, and arachnid mater tears, or complete root avulsions.

A high index of suspicion and severe trauma is required to diagnose traumatic avulsion pseudomeningocele of the brachial plexus. Clinical symptoms include headaches, paralysis, sensory deficits, and neuropathic pain. Low CSF pressure migraines are caused by meningeal irritation in the CSF, or pseudomeningocele, resulting from avulsed nerve roots. Around 15.2% of patients with traumatic brachial plexus injuries cause headaches [5].Pseudomeningocele is generally asymptomatic but is sometimes associated with a spinal hernia. Radicular involvement resulting from a brachial plexus injury results in a permanent neurological deficit and complete limb paralysis. A clinical examination is essential as an early assessment shows neurological stability or deterioration and dictates the timing of surgery.

Imaging techniques like MRI remain the gold standard for evaluating brachial plexus injuries. They can help distinguish between preganglionic and postganglionic injuries [6]. It is essential to confirm the diagnosis and assess the severity of nerve root avulsion and meningeal cyst formation, which helps determine the prognosis for these injuries [7]. Nerves are identified by MRI as linear, hypointense structures on T1 and T2 that are surrounded by fat. In trauma, a nerve injury is associated with a hematoma or pseudomeningocele. Pseudomeningocele is not an absolute indicator of root avulsion on MRI due to its high false-positive rate; however, it is correlated with clinical examination to enhance diagnostic accuracy [8].The association of MRI with electroneuromyography allows for determining the site and mechanism of the lesion. Therefore, MRI and electroneuromyography are complementary in assessing the reversibility of the brachial plexus lesions and choosing the most appropriate therapy. Unfortunately, MRI cannot differentiate precisely between pre-ganglionic and postganglionic nerve injury levels, which would be helpful in treatment. Surgical interventions are crucial to the management plan for traumatic avulsion pseudomeningocele. One of these cases shows significant clinical improvement following surgical methods, which include repair of the pseudomeningocele by suturing of the dural tear to stop leaks of CSF, followed by nerve grafting and nerve transfers, followed by regular physiotherapy. The positive results in this case highlight the significance of early surgical intervention and the possibility of substantial improvements in motor function and pain relief.

Novel procedures for pseudomeningocele repairs, like endovascular vessel occlusion using micro-coils and embolizing agents, have been documented in the literature. Treatment of patients with high-energy traumas should still follow the Advanced Trauma Life Support (ATLS) protocol and remain on cervical spine precautions. Clearance of the cervical spine is beyond the scope of this information; however, it is evident that when there is a ligamentous injury or in comatose patients, additional investigations in the form of dynamic cervical spine radiographs or an MRI are required [9]. After brachial plexus injuries, the main aim is to improve function. As the cells of the spinal cord's anterior horn remain, the prognosis for postganglionic injuries is favorable. If continuity is demonstrated during surgery, the likelihood of recovery increases. In patients with preganglionic injuries (root avulsions), nerve transfers are required because the cell bodies of native motor neurons have receded [10].

## Conclusions

In conclusion, this case series discusses the diagnosis, treatment, and prognosis of individuals with traumatic avulsion pseudomeningocele of the brachial plexus in great detail. The comprehensive surgical procedures and rehabilitation programs described in this series provide hope for individuals with this challenging illness. To better comprehend traumatic avulsion pseudomeningocele and its treatment options, as well as to improve the lives of those who are affected, more study and collaboration between medical experts are required. In the assessment of traumatic brachial plexus injuries, there was significant discordance among clinicians regarding the significance of the MRI findings. We used our case as a vignette to illustrate the clinical clues and indications to suggest brachial plexus root avulsions with our images.

## References

[1] Moses JE, Bansal SK, Goyal D (2013). Herniation of spinal cord into nerve root avulsion pseudomeningocele: a rare cause of delayed progressive neurological deficit. Indian J Radiol Imaging.

[2] Faglioni W Jr, Siqueira MG, Martins RS, Heise CO, Foroni L (2014). The epidemiology of adult traumatic brachial plexus lesions in a large metropolis. Acta Neurochir (Wien).

[3] Thatte MR, Babhulkar S, Hiremath A (2013). Brachial plexus injury in adults: diagnosis and surgical treatment strategies. Ann Indian Acad Neurol.

[4] Caporrino FA, Moreira L, Moraes VY, Belloti JC, Gomes dos Santos JB, Faloppa F (2014). Brachial plexus injuries: diagnosis performance and reliability of everyday tools. Hand Surg.

[5] Hébert-Blouin MN, Mokri B, Shin AY, Bishop AT, Spinner RJ (2013). Cerebrospinal fluid volume-depletion headaches in patients with traumatic brachial plexus injury. J Neurosurg.

[6] Silbermann-Hoffman O, Teboul F (2013). Post-traumatic brachial plexus MRI in practice. Diagn Interv Imaging.

[7] Wade RG, Takwoingi Y, Wormald JC, Ridgway JP, Tanner S, Rankine JJ, Bourke G (2019). MRI for detecting root avulsions in traumatic adult brachial plexus injuries: a systematic review and meta-analysis of diagnostic accuracy. Radiology.

[8] Laohaprasitiporn P, Wongtrakul S, Vathana T, Limthongthang R, Songcharoen P (2018). Is pseudomeningocele an absolute sign of root avulsion brachial plexus injury?. J Hand Surg Asian Pac Vol.

[9] Kumar Y, Hayashi D (2016). Role of magnetic resonance imaging in acute spinal trauma: a pictorial review. BMC Musculoskelet Disord.

[10] Karalija A, Novikova LN, Orädd G, Wiberg M, Novikov LN (2016). Differentiation of pre- and postganglionic nerve injury using MRI of the spinal cord. PLoS One.

